# RUVBL1/2 Complex Regulates Pro-Inflammatory Responses in Macrophages *via* Regulating Histone H3K4 Trimethylation

**DOI:** 10.3389/fimmu.2021.679184

**Published:** 2021-06-04

**Authors:** Rui Zhang, Chris Y. Cheung, Sang-Uk Seo, Hang Liu, Lakhansing Pardeshi, Koon Ho Wong, Larry M. C. Chow, Mary P. Chau, Yixiang Wang, Ah Ra Lee, Woon Yong Kwon, Sheng Chen, Bill Kwan-wai Chan, Kenneth Wong, Richard K. W. Choy, Ben C. B. Ko

**Affiliations:** ^1^Department of Applied Biology and Chemical Technology, The Hong Kong Polytechnic University, Hong Kong, China; ^2^State Key Laboratory of Chemical Biology and Drug Discovery, The Hong Kong Polytechnic University, Hong Kong, China; ^3^Department of Microbiology, Department of Biomedicine & Health Sciences, College of Medicine, The Catholic University of Korea, Seoul, South Korea; ^4^The University Research Facility in Chemical and Environmental Analysis, The Hong Kong Polytechnic University, Hong Kong, China; ^5^Faculty of Health Sciences, University of Macau, Avenida da Universidade, Taipa, Macau; ^6^Genomics and Bioinformatics Core, Faculty of Health Sciences, University of Macau, Avenida da Universidade, Taipa, Macau; ^7^Institute of Translational Medicine, Faculty of Health Sciences, University of Macau, Avenida da Universidade, Taipa, Macau; ^8^Department of Emergency Medicine, Seoul National University College of Medicine, Seoul, South Korea; ^9^Department of Infectious Diseases and Public Health, The City University of Hong Kong, Hong Kong, China; ^10^Department of Obstetrics and Gynaecology, The Chinese University of Hong Kong, Hong Kong, China

**Keywords:** RUVBL1/2, pro-inflammatory, macrophages, epigenetic modulation, H3K4 trimethylation

## Abstract

Macrophages play an important role in the host defense mechanism. In response to infection, macrophages activate a genetic program of pro-inflammatory response to kill any invading pathogen, and initiate an adaptive immune response. We have identified RUVBL2 - an ATP-binding protein belonging to the AAA+ (ATPase associated with diverse cellular activities) superfamily of ATPases - as a novel regulator in pro-inflammatory response of macrophages. Gene knockdown of *Ruvbl2*, or pharmacological inhibition of RUVBL1/2 activity, compromises type-2 nitric oxide synthase (*Nos2*) gene expression, nitric oxide production and anti-bacterial activity of mouse macrophages in response to lipopolysaccharides (LPS). RUVBL1/2 inhibitor similarly inhibits pro-inflammatory response in human monocytes, suggesting functional conservation of RUVBL1/2 in humans. Transcriptome analysis further revealed that major LPS-induced pro-inflammatory pathways in macrophages are regulated in a RUVBL1/2-dependent manner. Furthermore, RUVBL1/2 inhibition significantly reduced the level of histone H3K4me3 at the promoter region of *Nos2* and *Il6*, two prototypical pro-inflammatory genes, and diminished the recruitment of NF-kappaB to the corresponding enhancers. Our study reveals RUVBL1/2 as an integral component of macrophage pro-inflammatory responses through epigenetic regulations, and the therapeutic potentials of RUVBL1/2 inhibitors in the treatment of diseases caused by aberrant activation of pro-inflammatory pathways.

## Introduction

Macrophages are innate immune cells that play a central role in host defense against pathogens. They are also involved in tissue homeostasis, tissue repair, and disease pathogenesis. Among other functions, they sense invading pathogens and respond swiftly *via* induction of pro-inflammatory response characterized by the release of anti-microbial mediators including nitric oxide (NO), chemokines (CXCL8, CXCL10, CCL3, CCL4, and CCL5) and pro-informatory cytokines (IL-1 beta, IL-6, and TNF-alpha) respectively. These responses are triggered by the activation of toll-like receptors (TLRs) that recognize conserved microbial-associated molecular patterns of invading pathogens such as lipopolysaccharides (LPS), resulting in the activation of intracellular signaling cascades. Subsequently, there will be an increase in transcription of genes involved in the pro-inflammatory response (1), resulting in the direct elimination of invading pathogens, as well as activation of the adaptive immune response.

RUVBL1 (RVB1, TIP49, PONTIN) and RUVBL2 (REPTIN, RVB2, TIP48, TIP49B, and RBL1) are homologous members of the RuvB-Like family (2). They are ATP-binding proteins that belong to the AAA+ (ATPase associated with diverse cellular activities) superfamily of ATPases. RUVBL1 or RUVBL2, when expressed alone, displays no ATPase activity, but their ATPase activity can be significantly enhanced when they are assembled into a ring-like hetero-oligomeric complex (2, 3). RUVBL1 and RUVBL2 (RUVBL1/2) are involved in diverse cellular processes. They regulate transcription by modulating the transcriptional activities of MYC and β-catenin (4, 5), and act as a component of chromatin remodeling complexes TIP60, INO80, and SWR1, all of which regulates DNA damage response and chromatin remodeling (6–9). Besides genomic functions, they are also an integral component of the R2TP/Prefoldin-like cochaperone complex involved in the assembly of protein complexes including snoRNPs, RNA polymerase, telomerase, and members of the phosphatidylinositol 3-kinase-related protein kinase (PIKK) family (10).

Much less is known regarding the tissue-specific functions of RUVBL1/2. The human protein atlas showed that RUVBL1 and RUVBL2 are moderately expressed in immune cells (11). Concordantly, RUVBL2 is essential for T-cell development and T-cell dependent antibody responses (12), and is implicated in the differentiation of naïve CD4 T cells (13). Together, these data suggested that RUVBL1/2 play a role in immunity response. In addition, a role of RUVBL2 in metabolic homeostasis has been demonstrated, suggesting it as a potential therapeutic target for metabolic syndrome (14). On the other hand, a selective inhibitor of the RUVBL1/2 complex has been developed recently and showed efficacy as an experimental cancer therapeutics (15).

In this study, we investigated the functional role of RUVBL1/2 in innate immunity. We showed that RUVBL1/2 is indispensable for pro-inflammatory response and anti-microbial activity of macrophages. We further demonstrated that the expression of genes involved in pro-inflammatory response, including type-2 nitric oxide synthase (*Nos2*), are regulated in a RUVBL2-dependent manner *via* epigenetic regulations. Our study discovered a novel functional role of RUVBL1/2 in orchestrating innate immunity in macrophages, and highlighted the therapeutic potentials of RUVBL1/2 inhibitors in treating diseases caused by aberrant activation of pro-inflammatory pathways.

## Materials and Methods

### Reagents and Antibodies

Lipopolysaccharides (LPS) (*Escherichia coli* O111:B4) were purchased from Sigma Aldrich. ON-TARGETplus SMARTpool siRNA and single siRNA targeting mouse *Ruvbl2* and *Ruvbl1* were obtained from Dharmacon. For immunoblotting and ChIP assay, the following antibodies were used: mouse monoclonal anti-Reptin 52 (RUVBL2) (Santa Cruz), mouse anti-TIP49A (RUVBL1) (Abcam), mouse anti-β-Actin (Sigma-Aldrich), rabbit anti-p38/MAPK (Cell Signaling Technology), rabbit anti-p-p38/MAPK (T180/Y182; Cell Signaling Technology), rabbit anti-p44/42 (ERK1/2; Cell Signaling Technology), rabbit anti-p-p44/42 MAPK (ERK1/2; Thr202/Tyr204 (Cell Signaling Technology), rabbit anti-SAPK/JNK (Cell Signaling Technology), rabbit anti-p-SAPK/JNK (Thr183/Tyr185) (Cell Signaling Technology), rabbit anti-IκBα (Cell Signaling Technology), rabbit anti-Stat1 (Cell Signaling Technology), rabbit anti-p-Stat1(S727) (Cell Signaling Technology), rabbit anti-p-Akt (S473) (Cell Signaling Technology), mouse anti-LAMIN B1 (Santa Cruz), mouse anti-α-tubulin (Sigma-Aldrich), rabbit anit-p50 (Abcam), rabbit anti-H3K4Me3 (Cell Signaling Technology), rabbit anti-H4K20me3 (Santa Cruz Biotechnology), and rabbit anti-p50 (Cell Signaling Technology) antibodies. CB-6644 was obtained from MedChemExpress.

### Cell Lines and siRNA Transfection

RAW 264.7 macrophages (American Type Culture Collection) were grown in DMEM (Gibco), supplemented with 10% heat-inactivated fetal bovine serum (Gibco). Cells were transfected with the respective siRNA duplexes by using Neon™ transfection system (ThermoFisher) (1720V, 10ms, 2 pulses) or TransIn™ EL Transfection Reagent (Transgen Biotech). Cells were analyzed after two days of transfection.

### Preparation of Human Macrophages and LPS Stimulation of Human Monocytes

Human peripheral monocytes were isolated from the blood of healthy donors. Blood was diluted 2-fold with PBS. Mononuclear cells were isolated by gradient centrifugation with Pancoll human (PAN Biotech, Germany) following the manufacturer’s instructions. Monocytes were further isolated using EasySep Human Monocyte Isolation Kit (StemCell, Canada). The experiment has obtained approval from the institutional review board (IRB) of University College of Medicine/Seoul National University Hospital (IRB number: 1605-044-760), and written informed consent was obtained from the donors. Human monocytes were seeded at 2×10^5^ cells per well onto a 96 well plate and incubated at 37°C in 5% CO_2_. After stabilization, cells were washed with PBS without divalent ions. Cells were pre-treated with 1 µM of CB-6644 for 6 hours, followed by stimulation with LPS (10 ng/mL) for 6 hours in the presence of the same concentration of CB-6644, respectively. Supernatants were collected and spun at 1,500 rpm to remove floating cells. All samples were stored at -20°C before use.

### Measurement of Nitrite and Cytokines

NO produced by macrophages was measured using the Griess reaction system (Promega). Mouse IL-6, IL-1β, TNF-α, IFN-γ and GM-CSF level in the cultured medium was determined by Th1/Th2 Cytokine 11-Plex Mouse ProcartaPlex™ Panel according to the manufacturer’s recommendations (Invitrogen). Human IL-6 and TNF-α in culture medium was measured by ELISA MAX Standard Set Human IL-6 (BioLegend, USA) and TNF alpha Human Uncoated ELISA Kit (Invitrogen, USA), respectively, following manufacturer’s instructions.

### Macrophage Infection Assay

Macrophages infection assay was conducted as described (16). Specifically, RAW 264.7 macrophages were infected with *E. coli* DH5α with a multiplicity of infection of 10 for 1 h. Subsequently, cells were washed to remove extracellular bacteria. Gentamicin (100 µg/ml) was added to the medium for 1 h to prevent replication of the remaining extracellular bacteria, followed by the addition of gentamicin (25 µg/ml) until the end of the experiment. Infection was terminated by cellular lysis using 1% Triton X-100 in PBS, and the number of intracellular bacteria was determined by serial dilution in 0.05% Tween 20 in PBS and subsequent plating on LB plates. Colony-forming unit (CFU) were enumerated after incubation overnight at 37°C.

### RNA Isolation, Reverse Transcription, Real-Time PCR, and Relative Quantification

Total RNA from cell culture experiments was extracted with Trizol (Thermofisher) according to manufacturer’s instruction. RNAs were reverse transcribed using PrimeScript™ RT Master Mix (TaKaRa) and analyzed by real-time quantitative RT-PCR (qRT-PCR) on an ABI QuantStudio 7 Flex Real-Time PCR System (Applied Biosystems). Taqman Universal Mastermix and Assays-on-Demand (Applied Biosystems) were used to determine gene expression level. The following assays were used: *Ruvbl2* (Mm00600028_m1), *Ruvbl1* (Mm04203863_g1), *Actb* (Mm02619580_g1), *Ms4a7* (Mm01197655_m1), *Marcks* (Mm02524303_s1), *Il6* (Mm00446190_m1), *Csf3* (Mm00438334_m1), *Fpr2* (Mm00484464_s1), *Ccnd2* (Mm00438070_m1), *Vegfc* (Mm00437310_m1), *Csf2* (Mm01290062_m1), *Il1f6* (Mm00457645_m1), *Cxcl2* (Mm00436450_m1), *Siglec1* (Mm00488332_m1), *Lcn2* (Mm01324470_m1), *Ccr3* (Mm01216172_m1), *Acod1* (Mm01224532_m1), *Ccl6* (Mm01302419_m1), *Nos2* (Mm00440502_m1), *Tnfrsf1b* (Mm00441889_m1), *Ccl7* (Mm00443113_m1), *Il1b* (Mm00434228_m1), *Ccl2* (Mm00441242_m1), *Socs3* (Mm00545913_s1), *Ccl3* (Mm00441259_g1), *Ccl4* (Mm00443111_m1), *Car13* (Mm01291526_m1), *Pkm* (Mm00834102_gH), *Ccl5* (Mm01302427_m1), *Pck1* (Mm01247058_m1), *Il33* (Mm00505403_m1), *Mx1* (Mm00487796_m1), *Serpinb3b* (Mm03032256_uH), *Tnf* (Mm00443258_m1), *Ptgs2* (Mm00478374_m1). SYBR green qPCR was used for quantitation of other genes. Data were analyzed using the ΔΔCT method (relative to β-actin). The primers used are shown in **Supplementary Table 1**.

### RNA Sequencing and Bioinformatic Analysis

RNAs were prepared from RAW 264.7 cells under different treatments. RNA library construction and sequencing were conducted by Novogene. Raw reads were aligned to M. musculus reference genome (Genocode M25) using Hisat2 (17). Raw read counts for mouse genes were imported into DESeq2 using Bioconductor package tximport (version 1.16.1). Differential gene expression analysis was performed using Bioconductor package DESeq2 (version 1.28.1) (18). Default Benjamini & Hochberg method was used for multiple hypothesis correction of DESeq2 differentially expressed genes. Genes with q-value <= 0.05 and log2(fold-change) >= 1 or log2(fold-change) <= -1 were selected as significantly differentially expressed genes (DEGs). Gene Ontology enrichment was performed on significantly up- and down- regulated genes selected by the above cutoffs using the Bioconductor package topGO (version 2. 40.0). KEGG pathway enrichment analysis was performed using Bioconductor package KEGGprofile (version 1.30.0). All the raw sequencing data is deposited at NCBI SRA and can be accessed with accession IDs SRR13594158 - SRR13594165.

### Western Blot Analysis

Nuclear and cytoplasmic proteins were isolated using NE-PER Nuclear and Cytoplasmic Extraction Reagents kit (Thermo Fisher Scientific) according to instructions. Macrophages were washed twice with ice-cold PBS and lysed SDS lysis buffer (50 mM Tris-HCl, 2% SDS, 10% glycerol). The cell lysates were cleared by boiling. For immunoblotting, the proteins were resolved by SDS-PAGE, electrotransferred to either nitrocellulose membrane or polyvinylidene difluoride membranes and immunoblotted with antibodies. Anti-rabbit or anti-mouse horseradish peroxidase-linked IgG (GE Health) was used as secondary antibodies. Blots were developed with Luminata Forte Western HRP substrate (Millipore) and data were processed using Fluor-S MultiImager (Bio-Rad).

### MTS Assay

To determine cell viability, MTS assay was conducted using CellTiter 96 AQueous assay kit (Promega) according to manufacturer’s instructions. Macrophage were seeded in the 96-well plate. After gene knockdown or treatment with compound, freshly prepared MTS/PMS solution was added into each well, followed by incubation at 37°C, 5% CO_2_ incubator for 3 hrs. The absorbance at 490nm were measured with EnSpire Multimode Plate Reader (PerkinElmer).

### ChIP Analysis

Real-time PCR-based ChIP analysis was conducted as we have described previously (19). Cells were fixed with medium containing 0.9% formaldehyde for 15 min at room temperature. Sonicated chromatin fragments (averaged ~200 to 500 bp) were incubated with the corresponding antibody followed by pull-down using protein A agarose. Bound DNAs were eluted and quantitated by real-time PCR using corresponding primers. Primers used for PCR analysis were shown in **Supplementary Table 1**.

### Statistical Analysis

Statistical analysis was conducted using GraphPad Prism software. All data were expressed as mean ± SEM of triplicate experiments. Statistical significance was determined by unpaired t-test (two-tailed) for two groups or one-way ANOVA or two-way ANOVA for three or more groups comparison, followed by Bonferroni’s multiple comparison test as post-test. Significance is shown in the respective figures. p< 0.05 was considered statistically significant.

## Results

### *Ruvbl2* Is Important for Nitric Oxide Production and Bactericidal Activity of Macrophages

**Figure 1:**
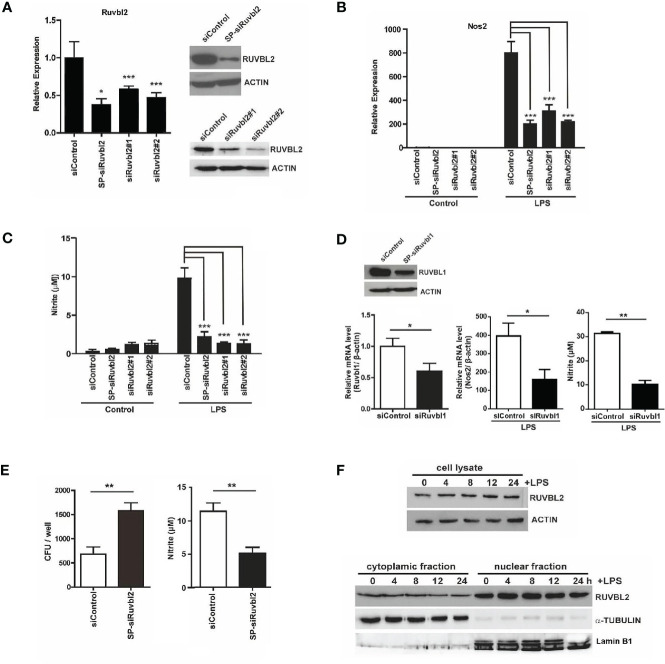
RUVBL2 is essential for *Nos2* gene expression and bactericidal activity of macrophages **(A)** Expression of *Ruvbl2* in RAW264.7 cells. Cells were transfected with SMARTpool siRNA (SP-siRuvbl2), individual siRNA (siRuvbl2#1, siRuvbl2#2), or control siRNA (siControl) for 48 hours. Left, RT-PCR analysis of relative *Ruvbl2* mRNA expression in response to different siRNA transfections. Right, Western blot analysis of RUVBL2 expression in cells transfected with siControl and SP-siRuvbl2 (upper panel), and with siControl, siRuvbl2#1 and siRuvbl2#2 respectively (lower panel). **(B)** Relative *Nos2* mRNA expression in RAW 264.7 cells transfected with the corresponding siRNAs in the presence or absence of LPS (10 ng/ml) for 24 hours. **(C)** Level of nitrite in culture medium of RAW 264.7 cells transfected with the corresponding siRNAs in the presence or absence of LPS (10 ng/ml) for 24 hours. **(D)** Left, expression of *Ruvbl1* gene in RAW 264.7 cells upon transfection with control siRNA (siRuvbl1) or siRNA against Ruvbl1 (siRuvbl1). Middle, level of *Nos2* expression in RAW 264.7 cells transfected with the corresponding siRNAs in the presence or absence of LPS (10 ng/ml) for 24 hours. Right, level of nitrite in culture medium of RAW 264.7 cells transfected with the corresponding siRNAs in the presence or absence of LPS (10 ng/ml) for 24 hours. **(E)** RAW 264.7 cells transfected with siControl or SP-siRuvbl2 were infected with *E.coli*. The level of bacterial load (left) and nitrite levels in the corresponding condition medium after 24 h (right) were determined. **(F)** Western blotting analysis showing the expression of RUVBL2 in whole cell lysate (Upper panel), and subcellular fractions (Lower panel), upon LPS treatment for different time points. Data from **(A–E)** were obtained from three independent experiments and presented in mean ± SEM. *p < 0.05; **p < 0.01; ***p < 0.001 by one-way ANOVA with Bonferroni’s multiple comparison test as post-test in **(A–C)** and by unpaired t-test in **(D, E)**.

To determine whether RUVBL2 plays a role in macrophage function, we conducted gene knockdown of *Ruvbl2* in mouse RAW 264.7 macrophages, using SMARTpool siRNAs (SP-siRuvbl2). The SP-siRuvbl2 effectively down-regulated *Ruvbl2* mRNA and RUVBL2 protein expressions in the cells, while no effect was observed when cells were transfected with non-targeting siRNA control (siControl) (**Figure 1A**). Knockdown of RUVBL2 did not compromise cell viability (**Supplementary Figure 1A**). Moreover, two additional independent siRNAs (siRuvbl2#1 and siRuvbl2#2) also demonstrated comparable efficiency to SP-siRuvbl2 for the knockdown of *Ruvbl2* mRNA and protein (**Figure 1A**). The role of RUVBL1 and RUVBL2 in pro-inflammatory response of macrophages was determined using *Nos2* expression response to LPS as an assay. LPS (10 ng/ml) treatment resulted in an 800-fold increase in *Nos2* expression in the siControl transfected mouse macrophages, whereas *Nos2* induction was significantly reduced in the cells transfected with SP-siRuvbl2 or individual siRNAs (siRuvbl2#1 and siRuvbl2#2) (**Figure 1B**). Concordantly, the concentration of LPS-induced nitric oxide (NO) production (measured as nitrite) was also significantly reduced in cells transfected with the different *Ruvbl2* siRNAs (**Figure 1C**). *Nos2* expression (**Supplementary Figure 1B**), and NO production (**Supplementary Figure 1C**) was similarly induced by LPS in untransfected and siControl-transfected macrophages, suggesting that the siControl siRNA does not disrupt pro-inflammatory response. It has been shown that RUVBL2 and RUVBL1 could either function independently, or together as hetero-dimeric or hetero-hexameric complex (20). Therefore, we determined the effects of gene knockdown of *Ruvbl1* on pro-inflammatory response. We found that gene knockdown of *Ruvbl1* (**Figure 1D**, left) resulted in significant reduction of both LPS-induced *Nos2* expression (**Figure 1D**, middle) and NO production (**Figure 1D**, right), suggesting that RUVBL2 works together with RUVBL1 in this context. Moreover, we found that gene knockdown of *Ruvbl2* also compromised the bactericidal activity of macrophages, as reflected by a remarkable increase in intracellular accumulation of *E. coli*. (**Figure 1E**, left), paralleled by reduced NO production (**Figure 1E**, right).

RUVBL2 has been shown to exert both cytoplasmic (14, 21) and nuclear activities (6–8). We therefore asked if LPS alters the expression and/or subcellular distribution of RUVBL2 using sub-cellular fractionation followed by Western blot analysis. Our data showed that neither expression (**Figure 1F**, upper) nor the subcellular distribution of RUVBL2 (**Figure 1F**, lower) was altered in the presence of LPS, suggesting that pro-inflammatory response of macrophage is regulated by a change in RUVBL1/2 activity. Together, these findings demonstrated that RUVBL2 plays a pivotal role in the innate immune response of macrophages.

### RUVBL2 Is an Essential Player in Pro-Inflammatory Response

**Figure 2:**
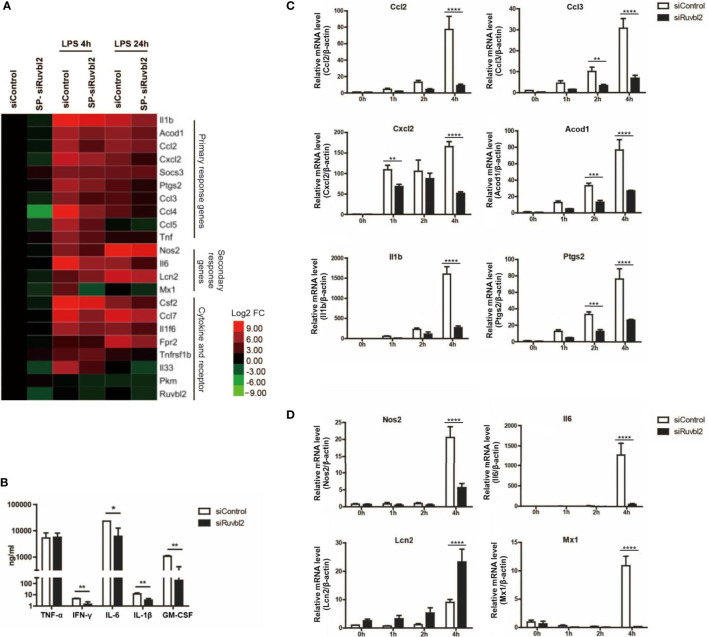
RUVBL2 is essential for pro-inflammatory gene expressions. **(A)** Heatmap of pro-inflammatory gene expression using real-time PCR analysis at 4 and 24 hrs after LPS induction of RAW 264.7 macrophages transfected with siControl or SP-siRuvbl2. **(B)** Level of TNF-α, IFNγ, IL-6, IL-1β, and GM-CSF in culture medium of RAW 264.7 macrophages transfected with siControl or siRuvbl2, in the presence of LPS (10 ng/ml). **(C)** Kinetics of expression of representative primary response genes in LPS (10 ng/ml)-induced RAW 264.7 macrophages transfected with siControl or SP-siRuvbl2. **(D)** Kinetics of representative secondary response genes expression in siControl and SP-siRuvbl2 transfected cells in response to LPS (10 ng/ml). Data from **(A–D)** are obtained from three independent experiments and presented in mean ± SEM. *p < 0.05; **p < 0.01; ***p < 0.001; ****p < 0.0001, by unpaired t-test in **(B)** and by two-way ANOVA with Bonferroni’s multiple comparison test as post-test in **(C, D)**.

Macrophages are crucial mediators of the pro-inflammatory responses. Upon stimulation by LPS, a complex transcriptional response is induced within hours, leading to the activation of functional programs that control cell migration, tissue repair and remodeling, antimicrobial defense and elucidation of adaptive immune response (22). Pro-inflammatory genes are categorized into primary and secondary response genes differentiated by their swiftness of upregulation upon TLRs stimulation (23). We selected twenty-two LPS-induced genes, including primary and secondary response genes, as well as cytokine and receptor genes, for analyzing their expression kinetics. Using real-time quantitative PCR analysis, we confirmed that these genes are significantly induced after 4 and 24 hours of LPS treatment (**Figure 2A**). There were differences in the kinetics of expression among different genes (**Figure 2A**). Comparing with cells transfected with siControl, transcriptional induction of *Nos2*, interleukin, interleukin-related genes (*Il1b, Il6*), prostaglandin-related (*Ptgs2*), and cytokines/chemokines (*Cxcl2, Ccl2, Ccl3, Ccl5, Csf2*) genes was reduced significantly at 4 or 24 hrs upon LPS treatment in macrophages transfected with SP-siRuvbl2, whereas the expression of *Tnf* was not affected (**Figure 2A** and **Supplementary Table 2**). As a control, the expression of pyruvate kinase (*Pkm*), a house-keeping gene whose expression was not altered by LPS treatment, was not affected by siRuvbl2 knockdown (**Figure 2A**). Concordant with these observations, SP-siRuvbl2-transfected cells showed a significant reduction in secretory IL6, IL1β, and CSF2 levels upon LPS treatment, whereas TNFα secretion was not inhibited by the siRNA (**Figure 2B**), consistent with the result of gene expression analysis.

To further elucidate if RUVBL2 differentially regulates the expression of primary and secondary response genes, we determined the expression of some of these genes over a short induction period (0 to 4 hrs) upon LPS stimulation. Among the selected primary response genes, the expression of *Ccl2, Ccl3, Il1b, Acod1, Cxcl2*, and *Ptgs2* in *Ruvbl2*-depleted cells was significantly reduced at 4 hours post LPS induction (**Figure 2C**). Similarly, there is a significant reduction in secondary response genes at 4 hours post LPS induction, including *Nos2, Il6*, and *Mx1*, in macrophages transfected SP-siRuvbl2 (**Figure 2D**). An exception was found for the gene *Lcn2*, which showed enhanced expression in *Ruvbl2*-depleted cells in response to LPS (**Figure 2D**). Together, our data suggested that RUVBL2 is essential for eliciting proper macrophage pro-inflammatory response upon LPS stimulation.

### RUVBL2 Inhibitor Effectively Blocks LPS-Induced Pro-Inflammatory Response

**Figure 3:**
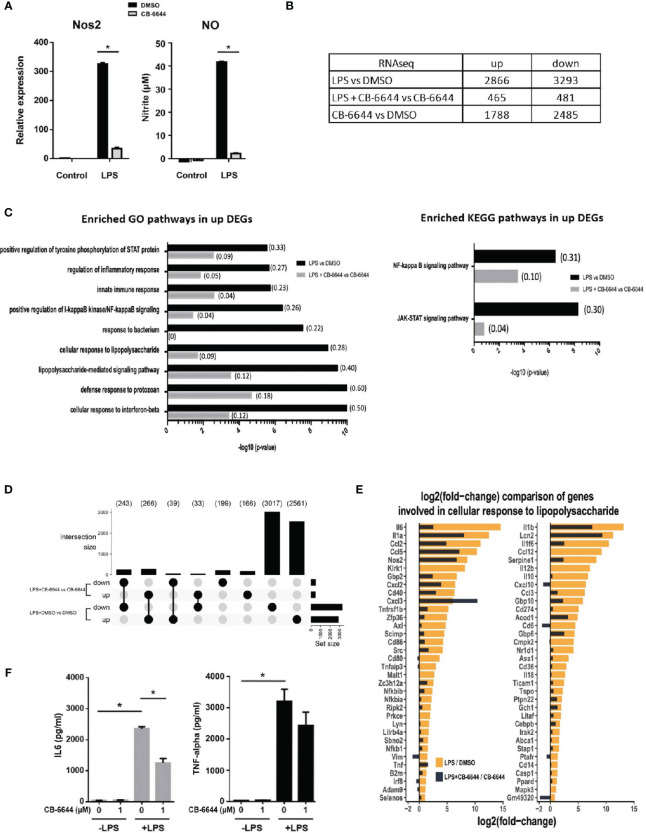
Transcriptomic analysis of pharmacological activity of CB-6644. Transcriptomic analysis of pharmacological activity of CB-6644. **(A)** Expression of *Nos2* (Left) and the level of nitrite in culture medium (Right) of RAW 264.7 macrophages induced with LPS (10 ng/ml) for 12 hours in the presence or absence of CB-6644 (1 μM). Data are presented in mean and +/- SEM of three independent experiments. *p < 0.0001 by one-way ANOVA test with Bonferroni’s multiple comparison test as post-test. **(B)** Number of up and down regulated genes from RNA-seq analysis in RAW 264.7 macrophages under different treatment. **(C)** Enriched GO and KEGG pathways in response to LPS treatment in the presence and absence of CB-6644 (1 μM). **(D)** Upset plot showing DEG comparison (> 2-fold, q-value < 0.05) of LPS-induced RAW 264.7 macrophages in the presence of DMSO or CB-6644 (1 μM) or RAW 264.7 macrophages in the presence of CB-6644 (1 μM). **(E)** Comparison of gene induction involved in GO term “Cellular Response to Lipopolysaccharide” by LPS treatment in the presence or absence of CB-6644 (1 μM). **(B–E)** are generated from the data of 2 independent experiments. **(F)** Level of IL-6 and TNF-α in culture medium of human monocytes induced with LPS (10 ng/ml) for 6 hours, in the presence of DMSO or CB-6644. Data are presented in mean and ± SEM of three independent experiments. *p< 0.0001 by one-way ANOVA test with Bonferroni’s multiple comparison test as post-test.

CB-6644 is a newly discovered RUVB1/2 complex inhibitor that specifically targets ATPase activity of the complex at high potency (15). Its effect on pro-inflammatory responses of macrophages has never been evaluated. We found that CB-6644 when added at a final concentration of 1 μM to the culture medium, similar to Ruvbl2 siRNAs, profoundly repressed *Nos2* gene expression and NO production in RAW 264.7 cells (**Figure 3A**). This effect is not due to cell death under the inhibition of RUVBL1/2 complex function, as the inhibitor treatment did not significantly affect cell viability (**Supplementary Figure 1D**).

To evaluate the impact of the CB-6644 in physiology and the pro-inflammatory responses of macrophages, transcriptome analysis by RNA-seq was conducted on RAW 264.7 macrophages, or LPS-induced RAW 264.7 macrophages in the presence and absence of the inhibitor. Differentially expressed genes (DEGs) were identified using DESeq2 (**Supplementary Table 3**). Sample distance analysis (**Supplementary Figure 2A**) revealed that gene expression profiles of biological repeats of treatment (n=2) are highly correlated with a median R value of 0.999 (**Supplementary Figure 2A**). Principal component analysis (PCA) showed that biological repeats are generally more similar to each other than to different treatments (**Supplementary Figure 2B**). Differential gene expression analysis by DESeq2 between LPS- and DMSO-treated cells revealed a total of 6159 DEGs (2866 up-regulated and 3293 down-regulated) (**Figure 3B** and **Supplementary Table 3**), including the primary and secondary response genes examined in the RT-PCR analysis (**Supplementary Figure 2C**). The up-regulated DEGs were enriched in GO terms such as Cellular Response to Lipopolysaccharide, Response to Bacterium, Inflammatory Response, and Innate Immunity response, etc., as well as the NF-kappaB and JAK-STAT signaling KEGG pathways – the two prototypical pathways activated by LPS signaling (24) (**Figure 3C** and **Supplementary Table 4**). These results show that the RNA-seq data reliably captured the macrophage response to LPS.

Strikingly, in the presence of the RUVB1/2 inhibitor CB-6644, the LPS-induced transcriptional response of macrophages was significantly mitigated with the number of DEGs being 6-7 times less (465 up-regulated and 481 down-regulated; LPS + CB-6644 *vs* DMSO + CB-6644). Specifically, most of the genes differentially regulated in response to LPS (**Figure 3D**; 2561 and 3017 for up- and down-regulated genes) were not significantly changed in their expression (i.e., less than 2-fold) in CB-6644-treated macrophages upon LPS treatment (**Figure 3D**; LPS + CB-6644 *vs* CB-6644), suggesting that the RUVB1/2 inhibitor CB-6644 can suppress LPS response in macrophages. More importantly, genes involved in the GO term Cellular Response to LPS were also significantly diminished with RUVB1/2 inhibition (**Figure 3E**, **Supplementary Table 4**). Together these data suggested that pharmaceutical inhibition of RUVBL1/2 complex results in the repression of pro-inflammatory responses in macrophages.

To further elucidate the relevance of this observation in humans, we determined the effect of CB-6644 in human primary monocytes. In these monocytes, the secretion of IL-6 and TNF-α was also highly induced when stimulated with LPS. In the presence of CB-6644, the secretion of IL-6 was significantly reduced, whereas the secretion of TNF-α was not affected in human monocytes (**Figure 3F**). NO production was not measured because it is well known that in human monocytes Nos2 gene is not responsive to LPS treatment. Together these findings suggested the functional conservation of RUVBL1/2 complex in pro-inflammatory responses between mouse and human.

### Elucidating the Mechanism of RUVBL2-Mediated Pro-Inflammatory Responses

**Figure 4:**
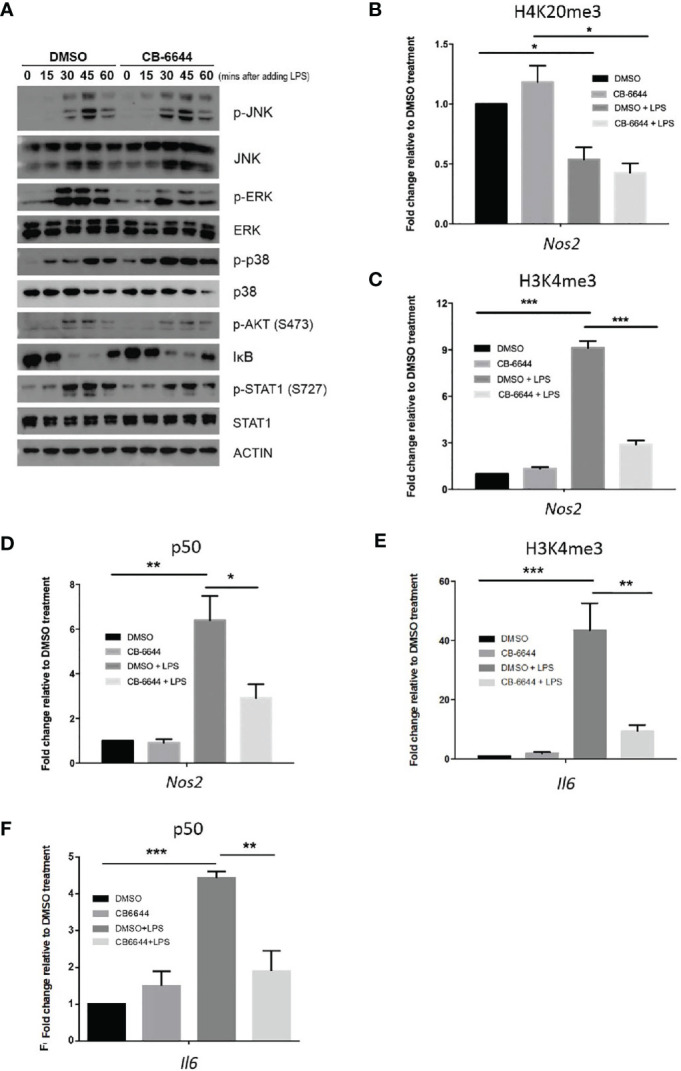
Mechanisms of RUVBL1/2 in the regulation of pro-inflammatory gene expressions. **(A)** Western blotting analysis of signaling molecules involved in TLR4 signaling pathways in the presence or absence of CB-6644. RAW 264.7 macrophages were induced with LPS (10 ng/ml) in the presence of DMSO or CB-6644 (1 μM) for the indicated time. Cell lysates were analyzed using the indicated antibodies. p-JNK, phospho-c-Jun N-terminal kinase; JNK, c-Jun N-terminal kinase; p-ERK, phospho-mitogen-activated protein kinase1/2; ERK, mitogen-activated protein kinase1/2; p-P38, phospho-P38; IκBα, NF-kappa-B inhibitor alpha; p-Stat1 (S727), phospho-Stat1 (S727); and Stat1. **(B–D)** ChIP-PCR analysis showing relative level of **(B)** H4K20me3, **(C)** H3K4me3, and **(D)** p50, around TSS of *Nos2* promoter in response to LPS (10 ng/ml), in the presence or absence of CB-6644. **(E, F)** ChIP-PCR analysis showing relative level of **(E)** H3K4me3 and **(F)** p50 around TSS of *Il6* promoter in response to LPS (10 ng/ml), in the presence or absence of CB-6644. For all ChIP-PCR analysis, cells were stimulated with LPS for 6 hours in the presence of CB-6644 (1 μM) or DMSO. Data represent fold enrichment in chromatin immunoprecipitated by the corresponding antibody relative to DMSO control. Data from **(B–F)** are presented in mean ± SEM of three independent experiments. *p < 0.05; **p < 0.01; ***p < 0.001, by one-way ANOVA with Bonferroni’s multiple comparison test as post-test.

LPS initiates sequential intracellular signaling events that lead to the expression of pro-inflammatory cytokines and mediators, primarily *via* Toll-like receptor-4 (TLR4) activation (22). Upon activation, TLR4 initiates an ordered recruitment of adaptor molecules, including MyD88, interleukin-1 (IL-1) receptor-associated kinase, and tumor necrosis factor (TNF) receptor-associated factor, leading to the activation NF-κB and MAPKS. Because RUVBL1/2 complex has been shown to play a role in cytoplasmic signal transduction and NF-κB activation (25, 26), we determined if RUVBL2 inhibitor impedes the activation of the known signaling pathways upon LPS stimulation. Stimulation of RAW 264.7 macrophages by LPS resulted in rapid phosphorylation of JNK, EKR, AKT, and p38, degradation of IκB, and phosphorylation of STAT1 (**Figure 4A**), consistent with established notions (22, 24). The addition of CB-6644 did not significantly inhibit these LPS-induced processes (i.e., activation of those signaling molecules) (**Figure 4A**). Together, these data suggested that RUVBL2 does not participate in major LPS-induced cytoplasmic signaling events. The negative effect of RUVBL1/2 complex on pro-inflammatory gene expressions was not mediated through affecting the activation NF-κB and STAT1 (i.e., IκB degradation and STAT1 phosphorylation, respectively), which are the major effectors of TLR4 signaling, suggesting that it may achieve gene regulation *via* epigenetic regulations.

Mounting evidence suggested that inducible pro-inflammatory response genes are maintained in a repressed state by corepressor complexes (27, 28) through trimethylation of H4K20 (H4K20me3) at gene promoters (29). Erasure of this histone mark by PHF2 is a pre-requisite for LPS-induced gene activation, including the *Nos2* gene, which is the target of PHF2 (29). We, therefore, conducted chromatin immunoprecipitation (ChIP)-PCR to elucidate if RUVBL1/2 complex regulates histone demethylations in *Nos2* promoter. Using primer pair located near κB enhancer and transcription start site (TSS) of *Nos2*, we found that H4K20me3 level was enriched around this region under basal condition (**Figure 4B**). LPS stimulation resulted in a consistent and significant reduction of H4K20me3 level at *Nos2* promoter (**Figure 4B**). Nevertheless, the addition of CB-6644 did not significantly affect basal level of H4K20me3, nor altered its erasure by LPS (**Figure 4B**), suggesting that RUVBL1/2 complex does not play a role in H4K20me3 demethylation nor gene depression during LPS stimulation.

On the other hand, pro-inflammatory gene activation is closely associated with H3K4 trimethylation (H3K4me3) around the TSSs of these genes (30). Using ChIP-PCR analysis, we found that H3K4me3 at the promoter of the *Nos2* gene were profoundly enriched by LPS stimulation, but the induction level was significantly reduced in the presence of CB-6644 (**Figure 4C**). ChIP-PCR analysis was then conducted to determine if the recruitment of NF-κB (as measured by the occupancy of p50) to the κB enhancer of *Nos2*, was regulated by RUVBL1/2. We found that p50 was significantly enriched at around κB enhancer of *Nos2* by LPS (**Figure 4D**). However, the association of p50 to the enhancer was mitigated by CB-6644 (**Figure 4D**), suggesting that RUVBL1/2 complex regulates NF-κB recruitment to *Nos2* promoter. Similarly, using primer pair located near the TSS of another major pro-inflammatory gene *Il6*, we found that CB-6644 also represses LPS-induced enhancement of H3K4me3 (**Figure 4E**). Furthermore, we found that LPS-induced enhancement of p50 to the *Il6* promoter is also inhibited by CB-6644 treatment (**Figure 4F**). Together, these data suggested that RUVBL1/2 complex regulates pro-inflammatory gene expressions through regulating H3K4me3.

## Discussion

RUVBL1/2 complex has been implicated in many cellular, physiological, as well as pathogenic processes, ranging from energy metabolism (25), glucose and lipid homeostasis (14), DNA repair (31), transcriptions (32), protein degradation (33), to cell growth and cancers (8, 34). Despite its broad involvement in various biological processes, the molecular functions of RUVBL1/2 complex remain largely elusive. Evidence suggested that upon undergoing heterodimerization into a hexameric or dodecameric ring structure, RUVBL1/2 exhibits enhanced DNA helicase activity that is linked to RNA polymerase activity (35, 36). However, RUVBL1/2 complex has also played a role in other protein complexes unrelated to its DNA helicase function, such as in the chromatin remodeling complexes (9, 37), telomerase (38), and phosphatidylinositol 3-kinase-related protein kinases (10). Besides, mounting evidence suggested that RUVBL1 and RUVLBL2 may work independently to elicit cellular response under specific context (12, 13, 39). More recently, the role of RUVBL1/2 complex as a chaperone has been proposed (37). Therefore, RUVBL1/2, either alone or in complex, may elicit cellular responses *via* diverse, yet uncharacterized mechanisms.

Here we presented multiple lines of evidence that RUVBL2 plays a novel functional role in the innate immune defense of human and mouse macrophages by mediating pro-inflammatory responses upon TLR activation. Our findings suggested that either depletion of RUVBL1 or RUVBL2 results in a similar inhibition of LPS-mediated *Nos2* gene expression and NO production, a hall mark in macrophages pro-inflammatory response which endows macrophages with cytostatic or cytotoxic activity against pathogens. Using a specific pharmacological inhibitor of RUVBL1/2 complex, we further demonstrated that the ATPase activity of the RUVBL1/2 complex is essential for the regulation of pro-inflammatory response of macrophages. Consistent with our finding, a recent CRISPR screen has also identified RUVBL2 as one of the putative regulators of LPS response in dendritic cells (40), substantiating our findings that RUVBL1/2 complex may serve broader functional roles in innate immunity.

TLR activation by LPS results in the activation of MyD88, NF-κB, and interferon regulatory factor signaling pathway, resulting in pro-inflammatory gene transcriptions (41). Besides being a nuclear protein, RUVBL1/2 is also localized to the cytoplasm participating in signal transduction events such as NF-κB activation (14, 21, 26). However, our data did not support a role of RUVBL1/2 in cytoplasmic signaling, because inhibition of RUVBL1/2 complex neither affects LPS-induced activation of major cytoplasmic signaling molecules (e.g., ERK, JNK, and p38) nor activates the central players in TLR signaling (NF-κB and STAT1). TLR activation leads to two waves of gene expressions categorized by the swiftness of transcriptional response, known as primary and secondary gene expressions respectively (23). Primary response gene expression, which does not require new protein synthesis, can be further divided into early primary (e.g., *Cxcl2, Socs3, IL1b, Ptgs2*) and late primary (e.g.*, Ccl2, Ccl5, Irg1*) response genes. Early primary response genes do not require nucleosome remodeling, whereas late primary response genes, and most (e.g., *Nos2, Il6, Lcn2, Mx1*) secondary response genes, require SWI/SNF-mediated nucleosome remodeling for activation (23). RUVBL1/2 has been shown to play a role in chromatin remodeling by being a component of ATP-dependent chromatin remodeling complexes including INO80, SWR1, SNF2-related CBP activator protein (SRCAP), TIP60 acetyltransferase complex (42), and BAF53, a component of the mammalian SWI/SNF-related protein complex (43). Nevertheless, our data showed that primary and secondary response genes were broadly disrupted to a similar extent upon inhibition of RUVBL1/2 complex. Therefore, our data suggest the role of RUVBL1/2 in the regulation of LPS-induced gene expressions is not acting *via* the SWI/SNF complex. On the other hand, although RUVBL1/2 has also been implicated in the regulation of RNA polymerase II activity (44), our transcriptomic analysis showed that RUVBL1/2 inhibition only selectively inhibits pro-inflammatory gene expressions rather than ameliorating global gene expressions. Therefore, it is unlikely that the complex regulates general transcription machinery *via* RNA Pol II. RUVBL1 or RUVBL2 has also been known to indirectly regulate gene transcriptions *via* cooperating with transcription factors such as Myc, E2F1 and *β*-catenin, respectively (4, 5, 45). Whether RUVBL1/2 exerts its effect *via* modifying the activity of specific transcription factors, such as NF-κB and STAT1, remains to be further elucidated.

Our study discovered a novel functional role of RUVBL1/2 complex in epigenetic regulation of LPS-induced transcriptional response in macrophages. Mounting evidence has highlighted the importance of epigenetic modifications in eliciting pro-inflammatory responses. Genes of the pro-inflammatory response are in a repressed state characterized by enrichment of repressive epigenetic mark H4K20me3 at the transcriptional start sites (TSSs) (29). LPS stimulation resulted in a NF-κB-dependent recruitment of histone demethylase PHF2 to these promoters, leading to the erasure of H4K20me3 (29). Activation of these genes is associated with concomitant increase of H3K4me3 at the TSS (46, 47). We showed that RUVBL1/2 complex has a precise role in histone methylations. The erasure of H4K20me3 in the TSS of *Nos2*, which is required for gene activation, was not significantly affected. Rather, we showed for the first time that RUVLB1/2 regulates the trimethylation of H3K4me3, and the recruitment of NF-κB to the enhancer of *Nos2* and *Il6* promoter. Mounting evidence suggested that a primary response genes contain high basal level of H3K4me3, whereas H3K4me3 modification at secondary response genes was up-regulated only upon LPS stimulation (23). H3K4me3 landscape has been hypothesized to guide NF-κB binding to pro-inflammatory genes (48), as well as for induction of *Nos2* and *Il6* (49, 50). Therefore, we speculated that RUVBL1/2-dependent regulation of H3K4me3 might play a crucial role in guiding the recruitment of NF-κB, and possibly other transcription factors such as STATs, to their target sites, leading to pro-inflammatory gene expressions. We believed RUVBL1/2 complex inhibition resulted in a genome-wide inhibition of LPS-induced NF-κB and STATs recruitment, as shown by the differential gene expressions that KEGG pathways related to NF-κB and JAK-STAT signaling were highly inhibited in the presence of RUVBL1/2 complex inhibitor. Regulation of histone methylations is complex and remains largely elusive. In mammals, members from both SET1 (51) and SMYD (52) family exhibit H3K4 methyltransferase activity. For SET1 proteins, the histone methylation property of each homolog is further dependent on its association with specific subunits (53). Furthermore, H3K4me3 was found to be regulated by monoubiquitination of histone H2B (54). On the other hand, the level of H3K4me3 is also regulated by demethylation *via* JAR1D1 protein (55). Besides histone methylations, H3K4 methyltransferases, such as MLL1 (47), MLL4/WBP7 (46), and SET7/9 (56), are also essential for the transactivation of NF-κB target genes. Therefore, more works are required to identify the specific target of RUVBL1/2 complex in the histone H3K4 methylation process. Nevertheless, our studies have shown for the first time the involvement of RUVBL1/2 complex in the regulation of pro-inflammatory gene expressions. The functional role of the complex in the regulation of H3K4me3 level has never been reported in other studies. More importantly, we demonstrated that RUVBL1/2 complex is a novel druggable target for targeting pro-inflammatory reactions in both human and mouse macrophages. Excessive inflammatory response contributes to sepsis (57), and over production of pro-inflammatory mediators are closely associated with the development of a variety of diseases ranging from neurodegenerative diseases (58) to rheumatoid arthritis (59). Therefore, it will be highly desirable to elucidate if RUVBL1/2 inhibitors may offer therapeutic benefits to these conditions. Evaluating the efficacy of RUVBL1/2 inhibitors in animal disease models in which pro-inflammatory prevails will be the next step. Notably, RUVBL1/2 complex inhibitors are being developed as potential anti-cancer therapeutics (15). However, our findings raised caution over targeting RUVBL1/2 complex cancer patients, as the treatment may cause a higher propensity to infections due to a compromised innate immune response – a potentially deadly side effect for cancer patients whose immune system may have already been weakened.

## Data Availability Statement

The datasets presented in this study can be found in online repositories. The names of the repository/repositories and accession number(s) can be found in the article/**Supplementary Material**.

## Ethics Statement

The studies involving human participants were reviewed and approved by institutional review board (IRB) of University College of Medicine/Seoul National University Hospital. The patients/participants provided their written informed consent to participate in this study.

## Author Contributions

BK designed and supervised the study, acquired funding for the study and write up the manuscript. RZ, CC, and MC performed cell experiments (gene knockdowns, western blots, drug treatments, phenotypic measurements), conducted data analysis and interpretation of data. S-US, AL, and WK conducted human monocyte experiments. HL, LP, KW, RC, and YW took part in ChIP-PCR, transcriptome or bioinformatics analysis. SC and BKC conducted bacterial infection experiments. LC provided resources. All authors contributed to the article and approved the submitted version.

## Funding

This work was supported by RGC General Research Fund (15106417), PolyU internal grant (P0009343), Research Impact Funds (R5050-18 and R4015-19F), and Collaborative Research Fund Equipment Grant (C5012-15E).

## Conflict of Interest

The authors declare that the research was conducted in the absence of any commercial or financial relationships that could be construed as a potential conflict of interest.

## Supplementary Material

Supplementary Figure 1 **(A)** Cell viability of wild-type macrophages *vs* macrophages transfected with siControl or SP siRUVBL2 siRNA at 72 hours post-transfection, measured by MTS assay. **(B, C)** Level of LPS-induced *Nos2* expression **(B)** and nitrite production **(C)** in wild-type *vs* siControl siRNA-transfected macrophages. **(D)** Cell viability of untreated, DMSO and CB-6644 treated RAW 264.7 macrophages at 24 hours post-treatment, measured by with MTS assay. Data from **(A–D)** are presented in mean ± SEM of three independent experiments. “ns”, not significant, defined as p ≥ 0.05 by unpaired t-test in **(B, C)**, and by one-way ANOVA with Bonferroni’s multiple comparison test as post-test in **(A, D)**.

Supplementary Figure 2 RNAseq data QC. Regularized log (rlog) transformation was applied on the DESeq2 normalized read counts. **(A)** Euclidean distance was computed between all samples and resulting distance matrix was clustered using hclust method. Distance heatmap shows similarity between biological replicates. **(B)** Principal Component Analysis (PCA) plot generated using rlog counts shows biological replicates cluster together and treatment specific samples cluster separately. **(C)** Heatmap of selected pro-inflammatory gene expression by RNA-seq analysis after LPS induction of RAW 264.7 macrophages treated with DMSO or CB-6644.

Supplementary Figure 3 Mechanisms of RUVBL1/2 in the regulation of pro-inflammatory gene expressions. **(A–C)** ChIP-PCR analysis showing relative level of **(A)** H3K4me3, **(B)** p50, and **(D)** H4K20me, around TSS of *Tnfα* promoter in response to LPS (10 ng/ml), in the presence or absence of CB-6644. For all ChIP-PCR analysis, cells were stimulated with LPS for 6 hours in the presence of CB-6644 (1 μM) or DMSO. Data represent fold enrichment in chromatin immunoprecipitated by the corresponding antibody relative to DMSO control. Data from **(A–C)** are presented in mean ± SEM of three independent experiments. *, p < 0.05, by one-way ANOVA with Bonferroni’s multiple comparison test as post-test.

Supplementary Table 1 Primer pairs used for RT-qPCR and ChIP-PCR analysis.

Supplementary Table 2 Real-time quantitative PCR analysis of selected genes of siControl- and SP-siRuvbl2-transfected RAW 264.7 cells, in the presence or absence of LPS respectively. Gene expression was normalized by beta-actin gene expression and expressed as fold induction relative to the expression level in siControl-transfected cells.

Supplementary Table 3 DEGs of RAW 2647 macrophages stimulated with LPS in the presence and absence of CB-6644. **(A)** LPS *vs* DMSO, **(B)** LPS + CB-6644 *vs* CB-6644, and **(C)** CB-6644 *vs* DMSO.

Supplementary Table 4 GO and KEGG pathways enriched in LPS-treated RAW 2647 macrophages in the presence or absence of CB-6644. **(A)** GO pathways of LPS *vs* DMSO. **(B)** KEGG pathways of LPS *vs* DMSO. **(C)** GO pathways of LPS + CB-6644 *vs* CB-6644. **(D)** KEGG pathways of LPS + CB-6644 vs CB-6644.
