# Supplementary figures and images for: RUVBL1/2 Complex Regulates Pro-Inflammatory Responses in Macrophages via Regulating Histone H3K4 Trimethylation
Source: Front Immunol. 2021 Jun 4;12:679184. doi: 10.3389/fimmu.2021.679184 (PMC8282052; doi:10.3389/fimmu.2021.679184)

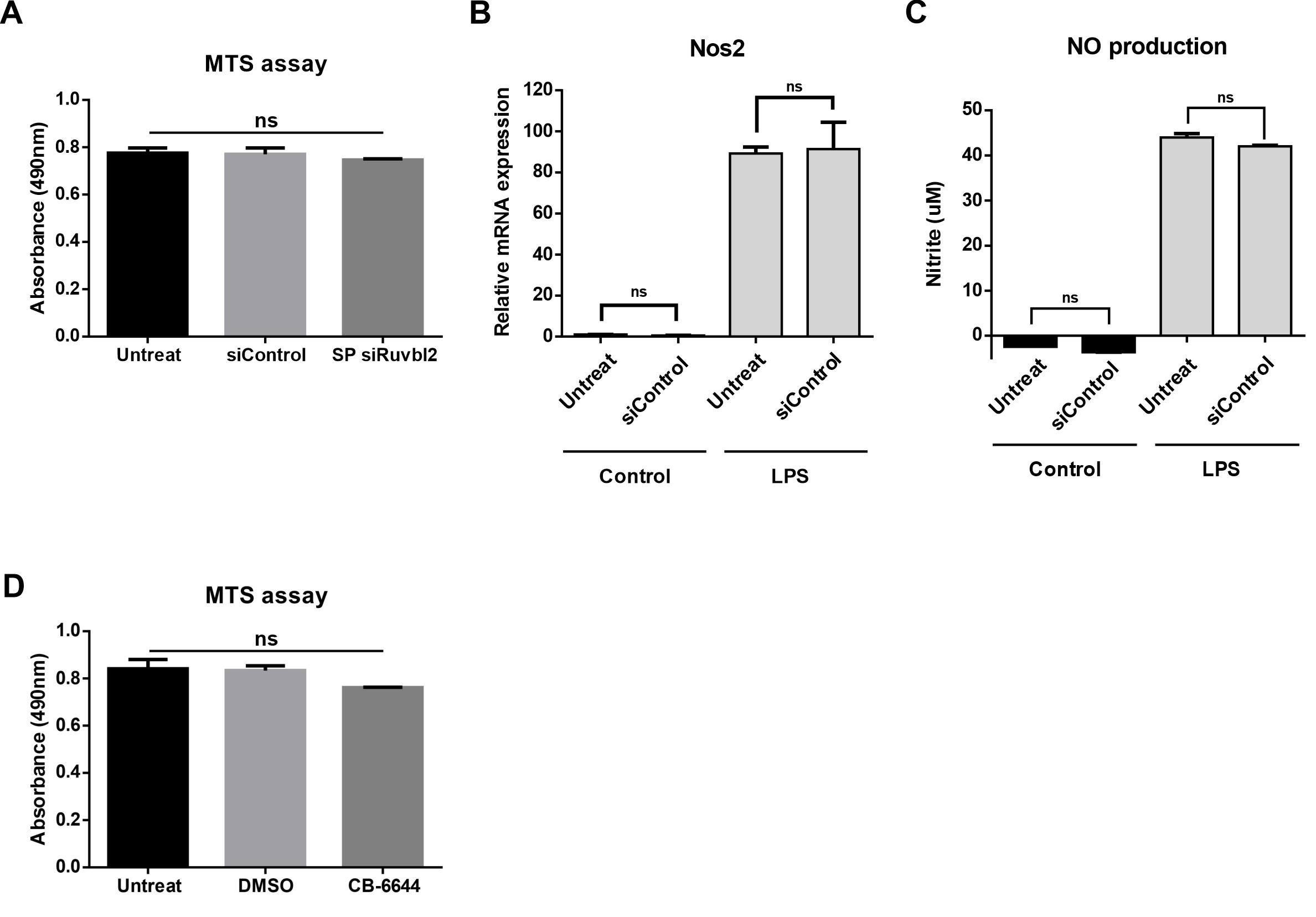

Supplement: Supplementary Figure 1 — (A) Cell viability of wild-type macrophages vs macrophages transfected with siControl or SP siRUVBL2 siRNA at 72 hours post-transfection, measured by MTS assay. (B, C) Level of LPS-induced Nos2 expression (B) and nitrite production (C) in wild-type vs siControl siRNA-transfected macrophages. (D) Cell viability of untreated, DMSO and CB-6644 treated RAW 264.7 macrophages at 24 hours post-treatment, measured by with MTS assay. Data from (A–D) are presented in mean ± SEM of three independent experiments. “ns”, not significant, defined as p ≥ 0.05 by unpaired t-test in (B, C), and by one-way ANOVA with Bonferroni’s multiple comparison test as post-test in (A, D). [file Image_1.tif]

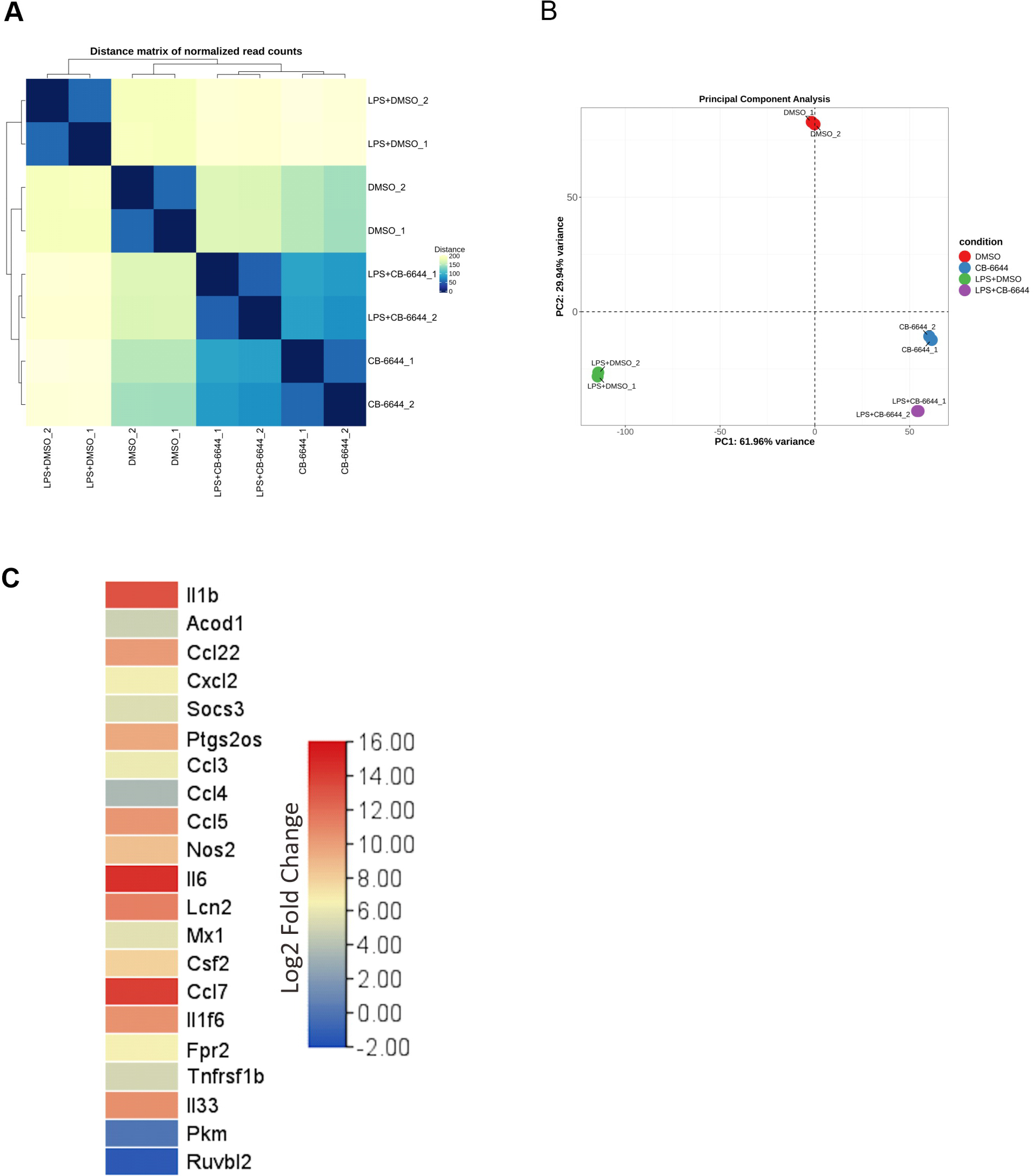

Supplement: Supplementary Figure 2 — RNAseq data QC. Regularized log (rlog) transformation was applied on the DESeq2 normalized read counts. (A) Euclidean distance was computed between all samples and resulting distance matrix was clustered using hclust method. Distance heatmap shows similarity between biological replicates. (B) Principal Component Analysis (PCA) plot generated using rlog counts shows biological replicates cluster together and treatment specific samples cluster separately. (C) Heatmap of selected pro-inflammatory gene expression by RNA-seq analysis after LPS induction of RAW 264.7 macrophages treated with DMSO or CB-6644. [file Image_2.tif]

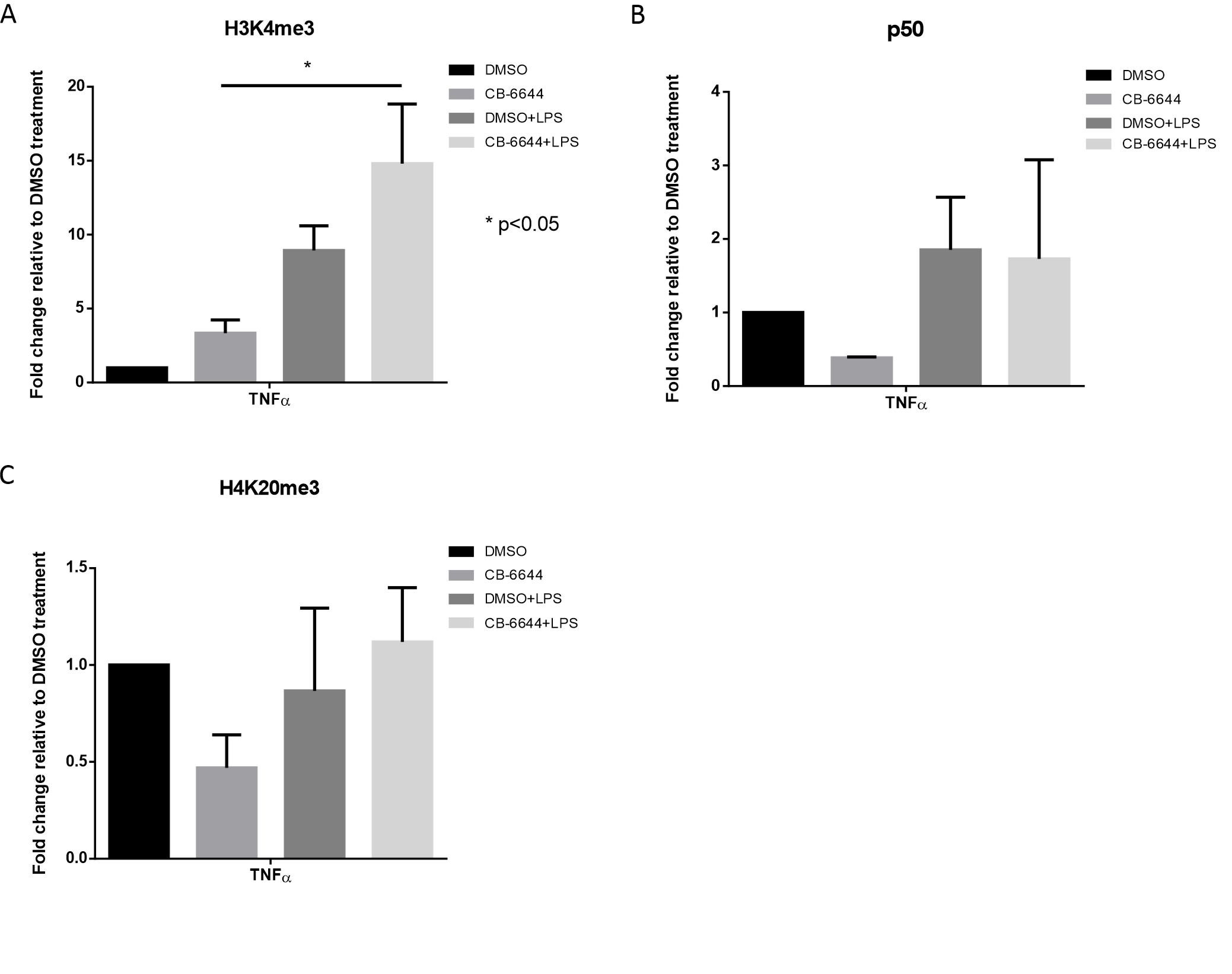

Supplement: Supplementary Figure 3 — Mechanisms of RUVBL1/2 in the regulation of pro-inflammatory gene expressions. (A–C) ChIP-PCR analysis showing relative level of (A) H3K4me3, (B) p50, and (D) H4K20me, around TSS of Tnfα promoter in response to LPS (10 ng/ml), in the presence or absence of CB-6644. For all ChIP-PCR analysis, cells were stimulated with LPS for 6 hours in the presence of CB-6644 (1 μM) or DMSO. Data represent fold enrichment in chromatin immunoprecipitated by the corresponding antibody relative to DMSO control. Data from (A–C) are presented in mean ± SEM of three independent experiments. *, p < 0.05, by one-way ANOVA with Bonferroni’s multiple comparison test as post-test. [file Image_3.tif]
