# Supplementary material for: RUVBL1/2 Complex Regulates Pro-Inflammatory Responses in Macrophages via Regulating Histone H3K4 Trimethylation
Source: Front Immunol. 2021 Jun 4;12:679184. doi: 10.3389/fimmu.2021.679184 (PMC8282052; doi:10.3389/fimmu.2021.679184)
Supplement: Supplementary Table 1 — Primer pairs used for RT-qPCR and ChIP-PCR analysis. [file Table_1.docx]

**Supplementary Table 1**

| Primers of RT-PCR | |
| --- | --- |
| mRuvbl2-F | 5’-ACCAAGATGATCGAGTCCCTG-3’ |
| mRuvbl2-R | 5’-ACCACCTCCTTGCGTTTCTG-3’ |
| mNOS2-F | 5’-CGGCAAACATGACTTCAGGC-3’ |
| mNOS2-R | 5’-TCGATGCACAACTGGGTGAA-3’ |
| mCxcl2-F | 5’-GCCAAGGGTTGACTTCAAGA-3’ |
| mCxcl2-R | 5’-ACTTTTTGACCGCCCTTGAG-3’ |
| mCcl3-F | 5’-AGATTCCACGCCAATTCATC-3’ |
| mCcl3-R | 5’-CCCAGGTCTCTTTGGAGTCA-3’ |
| mIl1b-F | 5’-GCTGAAAGCTCTCCACCTCA-3’ |
| mIl1b-R | 5’-AGGCCACAGGTATTTTGTCG-3’ |
| mCcl2-F | 5’-GGGCCTGCTGTTCACAGTT-3’ |
| mCcl2-R | 5’-GGGATCATCTTGCTGGTGAA-3’ |
| mLcn2-F | 5’-TTCCGGAGCGATCAGTTCC-3’ |
| mLcn2-R | 5’-TGACCAGGATGGAGGTGACA-3’ |
| mAcod1-F | 5’-CACAGAGAGCTTTGCTGGTATGA-3’ |
| mAcod1-R | 5’-TGCTCCTCCGAATGATACCAT-3’ |
| mPtgs2-F | 5’-CCCCTTCCTGCGAAGTTTA-3’ |
| mPtgs2-R | 5’-GAGAAGGCTTCCCAGCTTTT-3’ |
| mMx1-F | 5’-AAACCTGATCCGACTTCACTTCC-3’ |
| mMx1-R | 5’-TGATCGTCTTCAAGGTTTCCTTGT-3’ |
| mIl6-F | 5’-GTTCTCTGGGAAATCGTGGA-3’ |
| mIl6-R | 5’-TTTCTGCAAGTGCATCATCG-3’ |
| mActin-F | 5’-AGAGCTACGAGCTGCCTGAC-3’ |
| mActin-R | 5’-AGCACTGTGTTGGCGTACAG-3’ |
| Primers for ChIP-PCR | |
| NOS2 κB enhancer -F | 5’-AGGCAAGCACTTTACCAA-3’ |
| NOS2 κB enhancer-R | 5’-AGGGTCCACTCATCTTCG-3’ |
| IL-6-TATA-F (For H3K4me3) | 5’- CCCACCCTCCAACAAAGATT -3’ |
| IL-6-TATA-R (For H3K4me3) | 5’- ACTCCTCTCTCACAGTCTCAATA -3’ |
| IL-6-pro-F (for p50) | 5’- CACTTCACAAGTCGGAGGCT -3’ |
| IL-6-pro-R (for p50) | 5’- AATGAATGGACGCCCACACT -3’ |
